# Analysis of Feedback Mechanisms with Unknown Delay Using Sparse Multivariate Autoregressive Method

**DOI:** 10.1371/journal.pone.0131371

**Published:** 2015-08-07

**Authors:** Edward H. Ip, Qiang Zhang, Tomasz Sowinski, Sean L. Simpson

**Affiliations:** 1 Department of Biostatistical Sciences, Wake Forest School of Medicine, Winston-Salem, North Carolina, United States of America; 2 School of Information Sciences, University of Pittsburgh, Pennsylvania, United States of America; The University of Hong Kong, HONG KONG

## Abstract

This paper discusses the study of two interacting processes in which a feedback mechanism exists between the processes. The study was motivated by problems such as the circadian oscillation of gene expression where two interacting protein transcriptions form both negative and positive feedback loops with long delays to equilibrium. Traditionally, data of this type could be examined using autoregressive analysis. However, in circadian oscillation the order of an autoregressive model cannot be determined a priori. We propose a sparse multivariate autoregressive method that incorporates mixed linear effects into regression analysis, and uses a forward-backward greedy search algorithm to select non-zero entries in the regression coefficients, the number of which is constrained not to exceed a pre-specified number. A small simulation study provides preliminary evidence of the validity of the method. Besides the circadian oscillation example, an additional example of blood pressure variations using data from an intervention study is used to illustrate the method and the interpretation of the results obtained from the sparse matrix method. These applications demonstrate how sparse representation can be used for handling high dimensional variables that feature dynamic, reciprocal relationships.

## Introduction

In randomized clinical trials, multivariate longitudinal data are often sampled, either sparsely or densely (intensively) [1], over a certain time period. A large part of the longitudinal data analysis literature has been focused on the sparsely sampled data; e.g., data acquired by annual or semi-annual visits. For intensive longitudinal data, however, relatively fewer methods have been proposed, one important component of which is time series analysis [2]. The traditional time series approaches, such as univariate or multivariate autoregressive (AR) models, are only applied to one or several time series; e.g., a stock market index series or a commodity price series. However, in biological and clinical studies, we often observe one time series per subject, and in the multivariate case, data often shows a three-dimensional tensor structure, including the subject, the variable, and the time dimensions.

Jointly modeling multivariate intensive longitudinal data could introduce quite a few parameters. For example, the AR(*m*) model below:

$$Y_{ijt}=\sum_{\tau =1}^{m}\sum_{k=1}^{p}\rho_{kj\tau }Y_{ik,t-\tau }+\epsilon_{ijt},$$ (1)

would require *mp* ^2^ parameters. Here *Y*_*ijt*_ is the observed outcome of subject *i* at time *t* on variable *j*, *ρ*_*kjτ*_ is the contribution of the *k* ^*th*^ variables at time *t* − *τ* to the *j* ^*th*^ variable at time *t*, and the error term, *ϵ*_*ijt*_, is assumed to be independently and identically distributed (i.i.d.) with time-independent or stationary distribution assumption. Specifically, it can be assumed that $\epsilon_{ijt}∼\;N(0,\sigma_{j}^{2})$, and that *ϵ*_*ij*_1_*t*_1__ and *ϵ*_*ij*_2_*t*_2__ are independent if *j*_1_ ≠ *j*_2_ or *t*_1_ ≠ *t*_2_. We denote the number of variables as *p* and the order of the autoregression as *m*. The model specified by Eq (1) is a rather comprehensive model as it could include multiple possibly correlated variables, time-lagged effects from the same variable, as well as cross-lagged effects from all the other variables in the model. This kind of model has been found to be useful in applications such as fMRI time series analysis in which brain activities in various regions of the brain, intensively sampled over time, are modeled. For example, Harrison et. al, [3] used a multivariate AR model (p = 4, m = 3) for making inference about attention modulation of connectivity within the dorsal visual pathway and specifically across brain regions including the posterior parietal cortex and right prefrontal cortex. Therefore, it is possible that activity in the posterior parietal cortex at time *t* − 2 influences the right prefrontal cortex at time *t*.

Indiscriminatingly including all the variables and all time points as in Eq (1) is not always optimal especially when the sample size is small and overfitting problems often arise in such cases. Model selection criteria, such as Akaike Information Criterion (AIC), Bayesian Information Criterion (BIC), or other variations [4], would limit selection on the temporal component to the first few orders, but when the time period is long, one could miss significant autoregressive explanations from outcomes farther back in time. For example, daily blood pressure measurements often show strong correlations between hour 1 and hour 24. Only using measurements a few hours back would therefore miss the daily cycle. Another example is the circadian oscillations of gene expressions [5], where two interacting protein transcriptions can form both negative and positive feedback loops, with delays as long as 12 hours, while gene expression is measured hourly. These delays are essential to forming the periodic time series of protein densities, and trying to estimate these delays is an important step in understanding gene interactions on the molecular level. In terms of statistical modeling, neither an AR(1) nor an AR(12) are appropriate for these data because there exists only a few nonzero entries in the parameter set {*ρ*_*kjτ*_∣*k* = 1,…,*p*,*j* = 1,…,*p*,*τ* = 1,…,*T*}. This inspires a sparse autoregressive model, in which we only seek the first few most correlated autoregressive entries, regardless of the time lag or which variable. If we vectorize the parameter set into a vector, *ρ*, we would assume *ρ* is mostly zero except at a few entries. Some recent work on sparse autoregression models includes Fujita et. al. [6], who employ a multivariate AR model with *l*_1_ penalization to learn gene-regulatory mechanisms from time-course microarray data, and the Network Granger Causality (NGC) models of Lozano et. al. [7] and Basu et.al. [8] using group Lasso penality terms.

We often assume a time series has reached the equilibrium when samples are taken, but after an intervention, which could be time dependent—e.g., treatment dropped, switched, or with different dose levels given a subject’s conditions—we would like to know how the intervention alters the equilibrium. This is the case in our second motivating example, a multicenter randomized clinical trial in which hourly blood pressure data over a 24-hour period is recorded both before and after diet interventions. It is possible that after the interventions, equilibrium would reach a different state than before. This can be modeled through combining a linear mixed-effects (LME) model with an autogressive model [9], in which the LME model could include all the time dependent or independent predictors. Introducing random effects could also be beneficial, as subjects would often reach equilibrium differently, for example, depending on demographics or certain physiological characteristics.

Here we propose a sparse multivariate autoregressive analysis that takes into account the autocorrelations within the multiple observed outcomes over an arbitrarily long history, but only keeping those most correlated in the history. Hence while more variations can be explained, the model still remains parsimonious. We then combine the AR part with the LME part and jointly estimate both sets of parameters. The combined AR and LME model would specifically target time series that are often observed in clinical trials before and after intervention, which would be difficult to analyze using one sparse multivariate AR model, because an intervention often changes time series to a different equilibrium state.

## Motivating Examples

**Fig 1:**
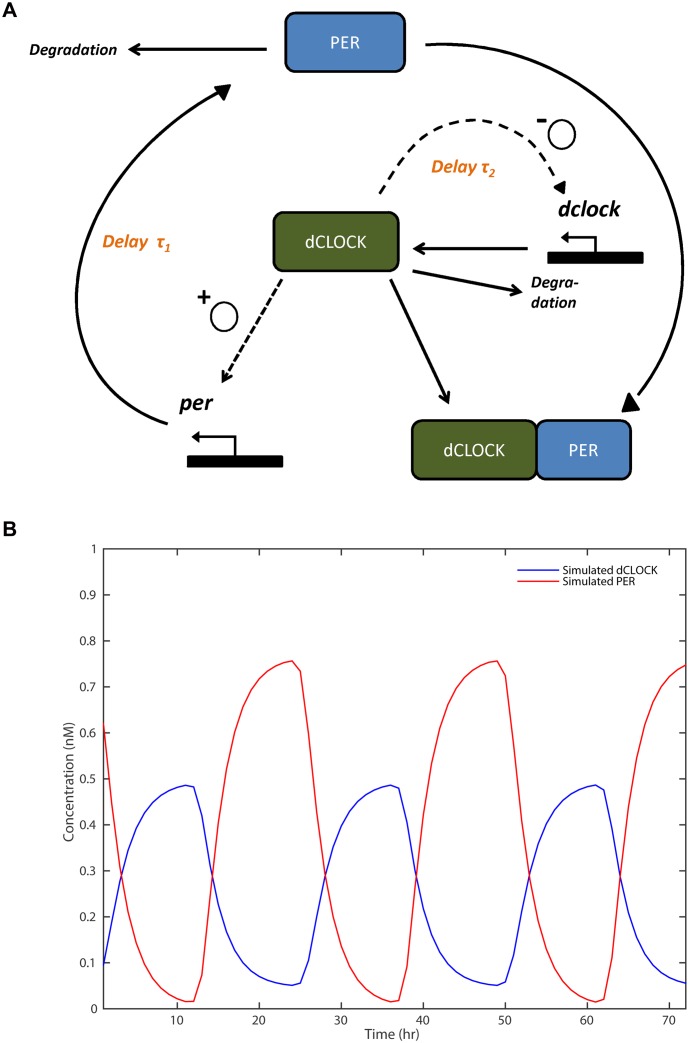
A simplified model (a) of *Drosophilia* circadian oscillator and (b) the output of the system as a function of time. Fig 1(b) is a rendition of Fig 1A in [5].

**Example 1**. Circadian rhythms reflect oscillating expressions of genes. Fig 1 schematically describes a simplified model of *Drosophilia* circadian oscillations [5], in which dCLOCK and PER represent two proteins while *dclock* and *per* represent their transcriptors respectively. The model contains both a positive and a negative feedback loop. Using dCLOCK protein level as an example, the two feedback loops work as follows: (1) dCLOCK activates *per* transcription and thus PER synthesis with lag *τ*_1_; PER binds with dCLOCK, decreasing the presence of dCLOCK (the negative feedback loop), and thereby also de-activates *per* transcription; and (2) increase in dCLOCK also leads to more dCLOCK (the positive feedback loop) because the activated PER binds to dCLOCK, leading to the de-repression of *dclock* transcription, with lag *τ*_2_. The two different lagged feedback mechanisms can be respectively modeled by eqs (2) and (3).

$$\frac{dY_{1}}{dt}=v_{1}\frac{1}{1+K_{1}+K_{1}e^{\alpha (Y_{1,t-\tau_{1}}-Y_{2,t-\tau_{1}})}}-k_{1}Y_{1},$$ (2)

$$\frac{dY_{2}}{dt}=v_{2}\frac{1}{1+{\left[K_{2}\left(1+e^{\alpha (Y_{2,t-\tau_{2}}-Y_{1,t-\tau_{2}})}\right)\right]}^{-1}}-k_{2}Y_{2},$$ (3)

where we use *Y*_1_ to denote PER and *Y*_2_ for dCLOCK. The model parameters, *K*_1_,*K*_2_,*v*_1_,*v*_2_,*k*_1_, and *k*_2_, are given as constants. The two time delays, *τ*_1_ and *τ*_2_, are essential to forming the circadian oscillations of *Y*_1_ and *Y*_2_. Eqs (2) and (3) are based on the ordinary differential equations in [5] with a slight modification. The quantity freely available dCLOCK protein Free dCLOCK was originally calculated by the function Free dCLOCK(*t*) = *max*([dCLOCK(*t*) − PER(*t*)],0). To avoid a possible discontinuity at zero in simulated data, we instead used the logistic transform exp(*αx*)/[1+exp(*αx*)], where *x* is Free dCLOCK(*t*) and *α* is a scaling parameter.

As we shall see later, the process can be approximated by the AR(*m*) model in Eq (1) in which an exponential transform exp(*Y*) replaces *Y* on the RHS of the equation. However, the traditional multivariate AR models would involve many unnecessary parameters, if, for example, the delays are long and/or more proteins are involved in the model; e.g., more complex ODE models in [10, 11]. It would be highly desirable if we could pinpoint the exact delays through an AR model but with nonzero entries only at certain delays. This example inspired our focus on sparsity.

**Example 2**. The Dietary Approaches to Stop Hypertension (DASH) trial was a multicenter, randomized parallel-arm feeding study that tested the effects of dietary patterns on blood pressure (BP). The three diets were a control diet (low in fruits, vegetables, and dairy products, with a fat content typical of the average diet in the United States), a diet rich in fruits and vegetables (a diet similar to the control except it provided more fruits and vegetables and fewer snacks and sweets), and a combination diet rich in fruits, vegetables, and low-fat dairy foods and reduced in saturated fat, total fat, and cholesterol (DASH diet). Participants were healthy adults 22 years of age or older who were not taking antihypertensive medication. The subjects’ BP measurements, including systolic blood pressure (SBP) and diastolic blood pressure (DBP), were taken over two 24-hour periods, one before the diet intervention and the other after. For more details, see [12] and [13].

After comparing the average BP (ABP) over a 24-hour period of cohorts before and after the intervention, Moore et. al. [13] found fruit/vegetable and DASH diets significantly (*p* < 0.0001) lowered ABP, when compared with the control diet (fruit/vegetable diet, -3.2/-1.0 mmHg; DASH Diet, -4.6/-2.6 mmHg). However, after considering within-subject correlation, the model by Simpson and Edwards [14] found the reduction in SBP by the DASH diet reduced from -4.6 mmHg to -3.6 mmHg. Presumably, the intervention altered the equilibrium of the BP cycles, and we can model this effect additively by adding intervention predictors onto the AR process. Because large BP variations are explained by previous measurements (the AR part), we expect a further reduction of the diet effects. Also, adding random effects would be useful for addressing subject-specific variations.

These two examples motivated us to combine a sparse multivate AR model with a linear mixed effects (LME) model to form a sparse multivariate autoregressive linear mixed effects model (SMARLME). The first example was used as a basis for simulations studies designed to determine how well our parsimonious model can accurately recover the original signal. We further analyzed the data from the second example to illustrate the utility of the model in a more traditional longitudinal data context. It is worth mentioning that the motivating example in [9]—i.e, parathyroid hormone (PTH) and serum calcium (Ca) levels interacting with the treatment Maxacalcitriol doze level—is also an excellent example for the SMARLME model. Compared to the AR(1) + LME model in [9], the SMARLME model could be more parsimonious and more far-reaching into the history of the interaction between PTH and Ca. These two examples demonstrate the flexibility of the SMARLME for modeling phenomena in which multi-variables in a system create feedback loops with specific lag times.

## Analysis

Let *Y*_*ijt*_ be the observed outcome of subject *i* at time *t* for variable *j*, and *X*_*iut*_ be the *u* ^*th*^ predictor for subject *i* at time *t*, *i* = 1,…,*N*, *j* = 1,…,*p*, and *t* = 1,…,*T*. The combined multivariate AR with the LME model can be described in scalar form as,

$$Y_{ijt}=\sum_{\tau =1}^{t-1}\sum_{k=1}^{q}\rho_{kj\tau }Y_{ik(t-\tau )}+\sum_{\tau =1}^{t-1}\sum_{u=1}^{r}\beta_{uj\tau }X_{iu(t-\tau )}+Z_{it}b_{i}+\epsilon_{ijt},$$ (4)

where vector *Y*_*ik*(*t* − *τ*)_ is the observed *k* ^*th*^ outcome of subject *i* at time *t* − *τ*, *ρ*_*kjτ*_ represents the contribution of the *k* ^*th*^ outcomes at time *t* − *τ* to the *j* ^*th*^ outcome at time *t*, and *β*_*ujτ*_ represents the contribution of the *u* ^*th*^ predictor at time *t* − *τ* to outcome *j*, and *X*_*iu*(*t* − *τ*)_ represents the value of the *u* ^*th*^ predictor. The terms *Z*_*it*_ and ***b***_*i*_ respectively represents the design matrix for the random effects and the vector of random effects. The simplest case would be *Z*_*it*_ being identity and ***b***_*i*_ being a single random effect *b*_*i*_ in which *b*_*i*_ is normally distributed with mean zero and variable *σ* ^2^. The error term, *ϵ*_*ijt*_, is assumed to be independent and normally distributed with constant variance, and independent from ***b***_*i*_. We denote the number of included predictive outcome variables by *q* (*q* ≤ *p*), and the number of predictors by *r*.

In contrast to the linear AR(*m*) model, this model is more flexible as well as comprehensive because it considers the entire history of observations of all variables including both outcomes and predictors. Furthermore, to accommodate a wider array of dynamical systems, transformed variable of *Y*_*ik*(*t* − *τ*)_ can be included as predictor. For example, for the circadian system described by the two ODEs in Eqs (2) and (3), we included exponentiated terms of *Y*_*ik*(*t* − *τ*)_ on the RHS of Eq (1). For the dynamic system in the circadian rhythm example, the nonlinear feedback mechanism would ensure stationarity of the model without necessarily constraining linear AR parameters, *ρ*. It is beyond the scope of this paper to discuss model stationarity, and we refer interested readers to [15].

In practical implementation of the model, we limit the history up to a certain period, *d*, such as a 24-hour period for observations with a strong daily cycle, and for shared parameters as in [14], we remove the variable index in *β*_*ujl*_ so that it becomes *β*_*ul*_. With the assumptions of equilibrium and time-independent *X*_*iu*_, we can further remove the time index and simply denote the regression parameter by *β*_*u*_.

The model specified by Eq (4) can be succinctly represented using matrix notation. To set up notation, we denote the vector (*Y*_*ijt*_, *j* = 1,…,*p*) by ***Y***_*it*_, and the *q* × *q* coefficient matrix (*ρ*_*kj*(*t* − *τ*)_,*k*,*j* = 1,…,*q*) at a given lag of *τ* by ***ρ***_*τ*_. Similarly, matrix ***β***_*τ*_ of size *p* × *r* is the coefficient matrix of ***X***_*i*(*t* − *τ*)_, where ***X***_*i*(*t* − *τ*)_ of size *r*×1 is the vector of predictors of subject *i*, and ***ϵ***_*it*_ is the vector (*ϵ*_*ijt*_) of length *p*.

In vector notation, the model now can be expressed as:

$$Y_{it}=\sum_{\tau =1}^{d}\rho_{\tau }Y_{i(t-\tau )}+\sum_{\tau =1}^{d}\beta_{\tau }X_{i(t-\tau )}+Z_{i}b_{i}+\epsilon_{it}.$$ (5)

The sparsity constraint is implemented through the following steps: (1) group all autoregression coefficients into a single vector—i.e., ***ρ*** = {*vec*(***ρ***_1_)^*T*^,*vec*(***ρ***_2_)^*T*^,…,*vec*(***ρ***_*d*_)^*T*^)^*T*^—and all predictor coefficients into a single vector —i.e., ***β*** = {*vec*(***β***_1_)^*T*^,*vec*(***β***_2_)^*T*^,…,*vec*(***β***_*d*_)^*T*^)^*T*^. Here *vec*(*A*) denotes the vector formed by vectorizing the *I* × *J* matrix *A* = (*a*_*ij*_) to form the vector (*a*_11_,*a*_21_,…,*a*_*I*1_,*a*_12_,…,*a*_*IJ*_)^*T*^. (2) Limit the number of nonzero entries in ***ρ*** and ***β*** to a given constant—i.e.,

$${\text{subject}\;\text{to}\;\|\rho \|}_{0}+{\left\|\beta \right\|}_{0}\leq n,$$ (6)

where ‖⋅‖_0_ is the *l*_0_ norm, or the number of non-zero entries in the vector. This implementation enforces sparsity in the set of the predictor coefficients when the predictors are time-varying and are not necessarily shared by all outcomes.

For a simpler form of the model, observe that the time-varying predictors, ***X***_*i*(*t* − *τ*)_, along with the predictors from which we seek sparse coefficients can be included into the AR part and treated as part of the outcome set, ***Y***_*i*(*t* − *τ*)_. Mathematically, the two forms are equivalent. Hence, we separate out the time-independent and shared predictors and simplify the model to:

$$Y_{it}=\sum_{\tau =1}^{d}\rho_{\tau }Y_{i(t-\tau )}+X_{i}^{T}\beta +Z_{i}b_{i}+\epsilon_{it},\;\text{subject}\;\text{to}\;{\left\|\rho \right\|}_{0}\leq n,$$ (7)

where vector ***β*** of size *r* × 1 is the shared time-homogeneous regression coefficient vector. The covariance structure of the error term, ***ϵ***_*it*_, is assumed to be conditionally independent given the other terms, including the fixed and random effects, in the model. We use the model specified in Eq (7) as the basic SMARLME model for subsequent discussions.

### Estimation method

Operationally, solving model Eq (7) involves both model selection and parameter estimation. We shall see that the proposed algorithm resolve the two problems jointly. To estimate the sparse *ρ* and *β* and the random effects, we take an alternating approach. In other words, we alternate between the estimation of the AR parameters and the fixed and random effects. First, given AR parameters ${\hat{\rho }}^{\left(s\right)}$ at the *s* ^*th*^ iteration, the model becomes a regular LME model with pseudo-outcomes, $Y_{it}^{†}=Y_{it}-\overset{}{\underset{}{\sum_{\tau =1}^{d}}}{\hat{\rho }}_{\tau }^{\left(s\right)}Y_{i(t-\tau )}$, and hence any LME estimating algorithm can be applied here with the independent covariance structure. The current estimates of the LME model can be used for the pseudo-outcomes, $Y_{it}^{*}=Y_{it}-X_{i}^{T}{\hat{\beta }}^{\left(s\right)}-Z_{i}{\hat{b}}_{i}^{\left(s\right)}$, where ${\hat{b}}_{i}^{\left(s\right)}$ is the predicted random effect vector, and can be used to solve the following sparse least-squares problem,

$$\min_{\rho }\sum_{i,t}\|Y_{it}^{*}-\sum_{\tau =1}^{d}\rho_{\tau }Y_{it-\tau }{\|}_{2}^{2},\text{subject}\;\text{to}{\left\|\rho \right\|}_{0}\leq n,$$ (8)

where ‖⋅‖_2_ is the *l*_2_ norm of vectors. Denote matrix $Y_{t}^{*}$ of size *N* × *p* as observations of all outcomes and all subjects at time *t*, and group all observations into a single vector—i.e., $y^{*}={\left(vec{\left(Y_{1}^{*}\right)}^{T},\ldots ,vec{\left(Y_{T}^{*}\right)}^{T}\right)}^{T}$. Similarly, vectorize *ρ*_*τ*_ into ***ρ***. After some matrix manipulations, we have the following *l*_0_ minimization problem,

$$\min_{\rho }\|y^{*}{-A\rho \|}_{2}^{2},\;\text{subject}\;\text{to}\;{\left\|\rho \right\|}_{0}\leq n.$$ (9)

For illustration purpose, we ignore the fixed and random effects. Matrix ***A*** of size *Np*(*T* − 1) × *p* ^2^ *d* has the following form,

$$A=(\begin{array}{ccccc} I_{p}⊗Y_{1}\\ I_{p}⊗Y_{2} & I_{p}⊗Y_{1}\\ I_{p}⊗Y_{3} & I_{p}⊗Y_{2} & I_{p}⊗Y_{1}\\ & .... & & & \\ I_{p}⊗Y_{t-1} & ... & I_{p}⊗Y_{t-d}\\ & .... & & & \\ I_{p}⊗Y_{T-1} & ... & I_{p}⊗Y_{T-d} \end{array}),$$ (10)

where square matrix ***I***_*p*_ of size *p* × *p* is the identity matrix, and ⊗ indicates the Kronecker product. An example of the *A* matrix and a practical refinement are given in S1 File.

The minimization problem in Eq (9) can be solved by a fast-computing Forward Backward greedy algorithm (FoBa), which we will briefly explain. For more details, see e.g., [16],[17]. The FoBa algorithm consists of two steps. The first step is forward searching. This step is equivalent to what statisticians call Forward Stepwise Regression or what signal processing researchers call Orthogonal Matching Pursuit [16]. See [18]. In this step, FoBa initializes a residual vector ***b*** = ***y***, the solution ***ρ*** = 0, and an index set **Γ** = ∅. At each iteration, it first finds the largest absolute entry *i* of the vector ***A*** ^*T*^ ***b***, and attaches it to **Γ**; i.e., **Γ** = **Γ** ∪ {*i*}. Next, it updates the solution entries in the index set **Γ** by solving ***b*** = ***A***_**Γ**_ ***ρ*_Γ_** through Gauss elimination, where ***A*_Γ_** represents a matrix with only columns of *A* in the index set Γ, and ***ρ*_Γ_** denotes the solution entries in Γ. Then it updates the residual vector, ***b*** = ***y*** − ***Aρ***, before the next iteration.

The step in seeking the largest absolute entry of ***A*** ^*T*^ ***b*** is equivalent to finding the most correlated column in *A* with *y*, before removing its contribution to *y* and moving on to search for the next most correlated. Conceptually, this is equivalent to seeking the most correlated ***Y***_*t* − *τ*_ with ***Y***_*t*_. Because there are only matrix-vector multiplications involved, the algorithm is very efficient. The procedure bears some apparent resemblance to the stepwise forward procedure in regression, which involves sequentially adding variable that improves the model the most in terms of criterion such as minimizing the residual sum of squares. Like the least square procedure typically used in stepwise forward selection, FoBa uses a greedy algorithm on the history of *Y* by sequentially searching for the next “best” variable. Thus the two approaches are similar in terms of their search strategy. However, they are also different in the following aspects: (1) the FoBa uses a selection procedure that is based on the largest inner product with the original elements in *A*, as opposed to based on the inner product with the normalized orthogonal elements in least square forward selection regression, and (2) a constraint is placed on the number of elements to be included in the selection set in FoBa, as opposed to stop adding variable according to a threshold of changing residual sum of squares in forward selection regression. The first point is subtle and carries computational implication: the FoBa only needs to orthogonalize the elements that are being selected whereas least square forward selection needs to orthogonalize all elements. See [19] for a detailed explanation.

The second step in FoBa is the backward step. It is designed to circumvent the problem that when an entry is chosen and included in **Γ**, it cannot be removed, thus implying that mistakes made in the early steps cannot be later corrected. The adaptive (FoBa) addresses this issue in the backward step [17]. At each iteration, FoBa searches through **Γ** to remove entries that would not significantly increase the least-square penalty term. The FoBa has shown to be a serious competitor to other algorithms for sparsifying matrices including LASSO [20][17]. Recently, other modifications in using the underlying orthogonal matching pursuit engine for finding a sparse solution to underdetermined systems of linear equation have been proposed, e.g., [18].

In terms of search strategy, the forward-backward approach in FoBa is analogous to forward-backward model selection in linear regressions, where significant variables are forwardly added in and then backwardly removed. Although the algorithm requires an input parameter, *n*, to restrict the number of nonzero entries in ***ρ***, the backward-search step would typically generate results with fewer nonzero entries in its solution. In other words, if the true model contains $\hat{n}$ nonzero entries, we can select *n* ≫ n*, and still be able to recover n* nonzero entries in ***ρ***. We illustrate this through our first example using a simulation study, which is described in the next section. In this sense, the model selection and estimation in the proposed SMARLME procedure can be jointly accomplished. In practice, we recommend a strategy of incrementing *n* in steps and select a model based on information criterion; e.g., Bayesian information criterion (BIC) or Akaike information criterion (AIC).

We summarize the estimation procedure as follows:

**Initialization**. Initialize ***ρ*** as ***ρ*** ^(0)^ = 0.

**Iterations**. At the *s* ^*th*^ iteration,

1. Given the current estimates ${\hat{\rho }}^{\left(s-1\right)}$, solve the linear mixed effects model through the pseudo-outcomes,
  $$Y_{it}^{†}=Y_{it}-\sum_{\tau =1}^{d}\rho_{\tau }^{\left(s-1\right)}Y_{it-\tau },$$ (11)
  and denote the current estimates of *β* as ${\hat{\beta }}^{\left(s\right)}$, and also estimate the predicted random effects ${\hat{b}}_{i}^{\left(s\right)}$.
2. Given $\hat{\beta }\left(s\right),{\hat{b}}^{\left(s\right)}$, update the pseudo-outcomes as
  $$Y_{it}^{*}=Y_{it}-X_{i}^{T}{\hat{\beta }}^{\left(s\right)}-Z_{i}{\hat{b}}_{i}^{\left(s\right)},$$ (12)
  and solve the *l*_0_ minimization problem stated in Eq (9) for ${\hat{\rho }}^{\left(s\right)}$, using the FoBa algorithm.

Note that if ***ρ*** = 0—i.e., the first step of the estimation procedure— we are solving a regular LME model without the AR part. The likelihood or the information criterion (AIC or BIC) of this model can be saved for later comparison with that of the SMARLME model for the justification of choosing the more complex SMARLME model.

## Results

Here we will present analysis results of two motivating examples, namely the circadian oscillator and BP measurements. The circadian-oscillator data were simulated using the ODEs in Eqs (2) and (3). The BP data set was a subset of data collected from the DASH study.

### 0.1 Simulation Studies: Circadian Oscillator

To facilitate simulation of data, we used the following discretized version of Eq (3) and Eq (3) by setting *dt* = 1. Here *Y*_1_ represents the variable PER, and *Y*_2_ represents the variable dCLOCK.

$$Y_{1}\left(t\right)-Y_{1}\left(t-1\right)=v_{1}\frac{1}{1+K_{1}+K_{1}e^{\alpha (Y_{1,t-\tau_{1}}-Y_{2,t-\tau_{1}})}}-k_{1}Y_{1}\left(t\right),$$ (13)

$$Y_{2}\left(t\right)-Y_{2}\left(t-1\right)=v_{2}\frac{1}{1+{\left[K_{2}1+e^{\alpha (Y_{2,t-\tau_{2}}-Y_{1,t-\tau_{2}})}\right]}^{-1}}-k_{2}Y_{2}\left(t\right),$$ (14)

**Fig 2:**
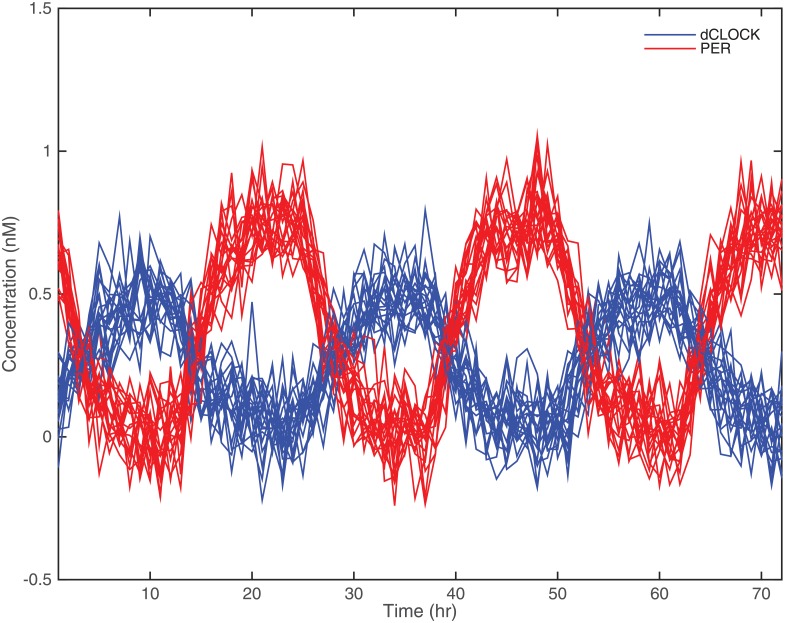
Sample of 100 simulated curves with added Gaussian noise of *σ* = 0.1.

We further set the two delays as *τ*_1_ = *τ*_2_ = 12, and set parameters in Eqs (2) and (3) as *v*_1_ = .5,*v*_2_ = .25,*k*_1_ = .5,*k*_2_ = .5,*K*_1_ = .3, *K*_2_ = .1, and *α* = 10. These values were based on values suggested by [5] and for offering realistic biological rhythms in the simulated data. Using Eqs (13) and (14), the true curves of simulated dCLOCK and PER over time, referred to as no-noise data hereafter, are shown in Fig 1(b). To simulate realistic data, Gaussian white noise of different levels was then added to the no-noise data. We choose three levels of Gaussian noise—i.e., *σ* = 0.01,0.05, and 0.1—and the simulation and estimation are repeated 1,000 times for each noise level. The simulated sample of 100 curves with added noise of standard deviation *σ* = 0.1 are shown in Fig 2 over a 72-hour period.

No time-independent and shared predictors are given in this simulation experiment; our sole purpose is to recover the most correlated entries in history with ***Y***_*t*_. In addition to having the linear terms of ***Y***_*t* − *τ*_ in the AR part, we also include exp***Y***_*t* − *τ*_ terms. To make the model as parsimonious as possible, we set the history period *d* = 15, three hours greater than *τ*_1_ and *τ*_2_, and the number of nonzero entries in *ρ* as *n* = 25. Using the no-noise data and the FoBa algorithm, we identified 7 nonzero locations. Thus using *n* = 25 is substantially larger than the true number of nonzeros, n* = 7. Setting *n* ≫ n* helps us justify whether the forward-backward greedy algorithm can successfully remove uncorrelated entries while keeping the most correlated entries.

**Fig 3:**
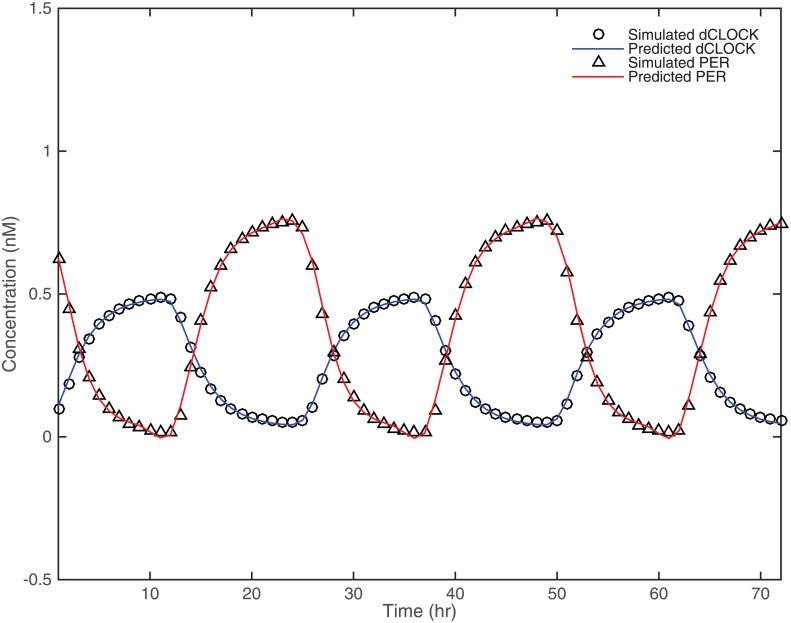
Observed values and fitted values based on estimates from FoBa for data without noise.

The FoBa algorithm applied to the no-noise data resulted in 7 non-zero coefficients in the linear model. The positions, indexes, and values for the nonzero terms predicting the system (***Y***_1_,***Y***_2_) are depicted in Table 1. The observed no-noise data and the predicted values based on the linear system with 7 non-zero entries are depicted in Fig 3. It can be seen that the recovery of the original curve is almost perfect when noise is not present.

**Fig 4:**
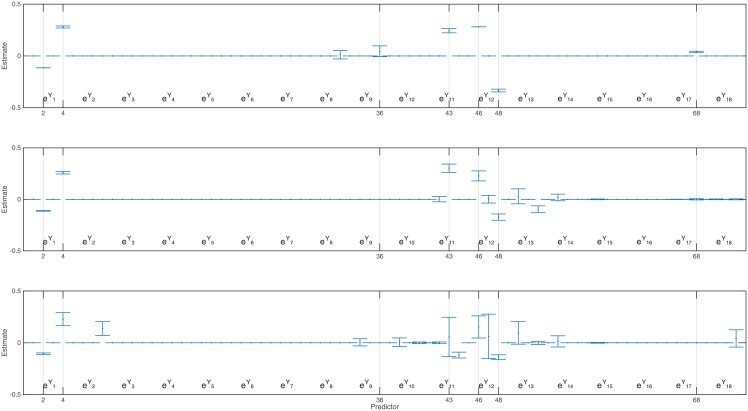
The mean and confidence interval of estimated *ρ*, shown as a single vector. The vertical lines represent the positions of true nonzero values. The three panels (top to bottom) respectively show results for three levels of noise: *σ* = 0.01,0.05,0.1.

Fig 4 shows the mean and confidence intervals (in error bars) of estimates derived from 1,000 replicates by applying FoBa to simulated data for each noise level. The AR parameters *ρ*_*kjτ*_ are organized as a single vector, with the first index changes fastest. The vertical lines correspond to the positions of which true non-zero terms are located. Because only exp***Y***_*t* − *τ*_ terms were selected, and none of the ***Y***_*t* − *τ*_ terms were chosen in any replication, Fig 4 does not include parameters for the ***Y***_*t* − *τ*_ terms.

**Fig 5:**
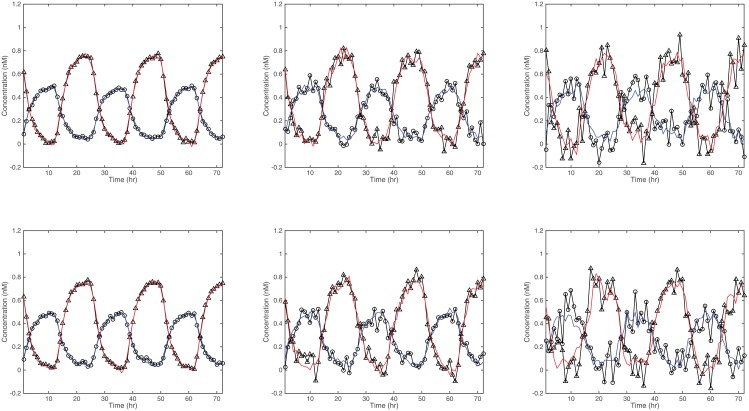
Observed and fitted values of two randomly selected samples (as rows) from each noise level *σ* = 0.01,0.05,0.1 respectively from the leftmost column to the rightmost column. The observed data are represented in triangles (PER) and circles (dCLOCK), and the lines represent fitted values.

Fig 5 shows the observed and predicted values of the variables *Y*_1_ and *Y*_2_ at the three designated levels of noise. Because of space, out of *n* = 1,000 samples we randomly selected two at each level to show how well the FoBa algorithm recovers the pattern. To further summarize the fits of the SMARLME model based on FoBa estimates, in Table 1 we present the results of the simulation study in the form of bias and mean squared error (MSE) of the estimates over 1,000 replications. Bias and MSE are defined as follows:

$$\text{Bias}=\frac{1}{NL}\sum_{n,l}\left|\hat{\rho_{l}^{\left(n\right)}}-\rho_{l}^{\left(n\right)}\right|,\text{MSE}=\frac{1}{NL}\sum_{n,l}{\left(\hat{\rho_{l}^{\left(n\right)}}-\rho_{l}^{\left(n\right)}\right)}^{2},$$ (15)

where $\rho_{l}^{\left(n\right)}$, *l* = 1,…,*L* denotes the AR parameter derived from the *n* ^*th*^ replication, where *n* = 1,…,*N*, and *N* was set at 1,000 in this experiment.

**Table 1:** Statistics on the Estimated Parameters.

Position	True value	*k* *	*j* *	*τ* *	Noise level	Count	Bias (SD)	MSE (SD)
2	-0.11474	1	2	1	0.01	1000	0.0001 (1.2E-04)	3.471E-08 (3.9E-08)
2	-0.11474	1	2	1	0.05	1000	0.0028 (1.6E-03)	1.019E-05 (8.4E-06)
2	-0.11474	1	2	1	0.1	1000	0.0092 (3.9E-03)	1.008E-04 (4.3E-05)
4	0.27467	1	1	1	0.01	1000	0.0050 (5.0E-03)	4.948E-05 (7.8E-05)
4	0.27467	1	1	1	0.05	1000	-0.0160 (6.0E-03)	2.916E-04 (1.1E-04)
4	0.27467	1	1	1	0.1	1000	-0.0469 (3.2E-02)	3.210E-03 (3.0E-03)
36	0.063997	1	1	9	0.01	753	-0.0184 (2.6E-02)	1.021E-03 (1.8E-03)
43	0.251	1	1	11	0.01	1000	-0.0077 (1.0E-02)	1.666E-04 (2.9E-04)
43	0.251	2	1	11	0.05	999	0.0517 (2.0E-02)	3.079E-03 (2.3E-03)
43	0.251	2	1	11	0.1	258	-0.1945 (9.6E-02)	4.703E-02 (2.7E-02)
46	0.28112	2	2	12	0.01	1000	-0.0002 (1.2E-04)	4.265E-08 (4.8E-08)
46	0.28112	1	2	12	0.05	1000	-0.0533 (2.5E-02)	3.449E-03 (3.1E-03)
46	0.28112	1	2	12	0.1	902	-0.1283 (5.4E-02)	1.936E-02 (2.0E-02)
48	-0.34163	1	1	12	0.01	1000	0.0082 (6.9E-03)	1.144E-04 (1.9E-04)
48	-0.34163	1	1	12	0.05	1000	0.1694 (1.5E-02)	2.893E-02 (4.0E-03)
48	-0.34163	1	1	12	0.1	997	0.2021 (1.1E-02)	4.099E-02 (5.3E-03)
68	0.03964	1	1	17	0.01	999	0.0001 (2.9E-03)	8.563E-06 (5.0E-05)
68	0.03964	1	1	17	0.05	8	-0.0394 (2.8E-03)	1.559E-03 (1.3E-04)

* k = predictor variable, j = estimated variable, *τ* = lag

In general, the SMARLME procedure recovers the parameters quite well, as evidenced by the small biases and mean squared errors. The result also shows that both bias and MSE increase with the level of noise in the data, as expected. We also make the following observations: (1) FoBa provides almost perfect fit to the nonlinear no-noise data using a small number of nonzero coefficients. The coefficients at lag 1 and 12 are substantial and are consistent with the way data were generated. (2) When *σ* = 0.1, and given that the true signal variance at 0.0268, we have a rather low signal-noise ratio (SNR) of 2.7, suggesting that the algorithm can recover true time lags reasonably well even under very noisy situations. Here SNR is defined as the signal variance divided by the noise variance. (3) There exist non-zero coefficients in locations that are not expected—e.g., at lag 11. This may arise because data at lag 11 are highly correlated with data at lag 12. An implication of this observation is that there potentially exist multiple solutions that fit the observed data equally well. (4) There exist some small coefficients which are close to zero—e.g., *ρ*_2,2,17_ at position 68. For this position, as the noise level increases, FoBa is less likely to select coefficient at this location. This is reflected in the Count column in Table 1, which represents the number of times that the model select the correct position of predictor out of 1,000 replications. At *σ* = 0.1, FoBa does not select this location at all. An implication of this observation is that for coefficients with small nonzero values, they are not always selected especially when the noise level is substantial. (5) Regardless of the model selected, the FoBa provides predicted values that fit the observed values quite well (see Fig 5). This simulation indeed demonstrates that the SMARLME could effectively recover intrinsic highly-correlated delays in periodic data with feedback loops.

### 0.2 Data Application: Blood Pressure Data

**Fig 6:**
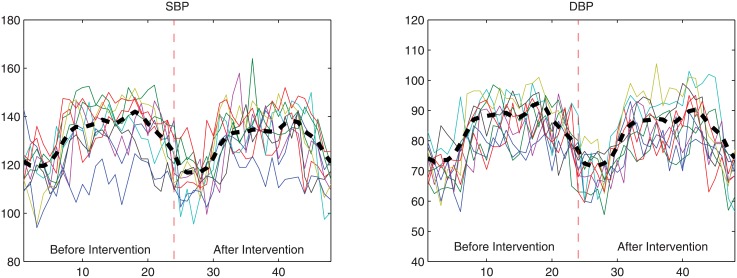
The 24-h BP data of a subsample of 50 subjects before and after intervention.

As noted in [14], more work is needed on the longitudinal analysis of 24-hour blood pressure data given the lack of a generally accepted ‘standard’ analysis method. Hence the appeal of illustrating our method with the DASH data. The 24-hour hourly BP data of a sample of 340 subjects before and after intervention is concatenated together to form a 48 x 1 vector for the SBP and the DBP of each subject. The SBP and DBP data of a subsample of 50 subjects, before and after intervention, are shown in Fig 6, along with the sample mean curves in thick, black dashed lines. An intervention variable, *δ*_*t*_, is introduced to differentiate the before from the after intervention period; i.e., *δ*_*t*_ = 0,1 ≤ *t* ≤ 24;*δ*_*t*_ = 1,25 ≤ *t* ≤ 48. Thus, our model accounts for the three week distance between the measurements before the intervention period and those after. Three diet groups are coded as two separate binary variables, namely the vegetable/fruit diet and the DASH diet. Eight predictors include intercept, vegetable/fruit diet, DASH diet, control diet and intervention period, vegetable/fruit diet and intervention period, DASH diet and intervention period, race, and age. The AR equilbria without intervention are assumed to be the same before and after intervention, and hence the intervention effects can be separately estimated. A subject-specific random effect is added onto the intercept term. We set *d* = 23, and the total number of entries in *ρ* is *p* ^2^ *d* = 2^2^×23 = 92. For model selection, we vary *n* from 0 to 59, and choose the minimum-BIC model within this range.

**Fig 7:**
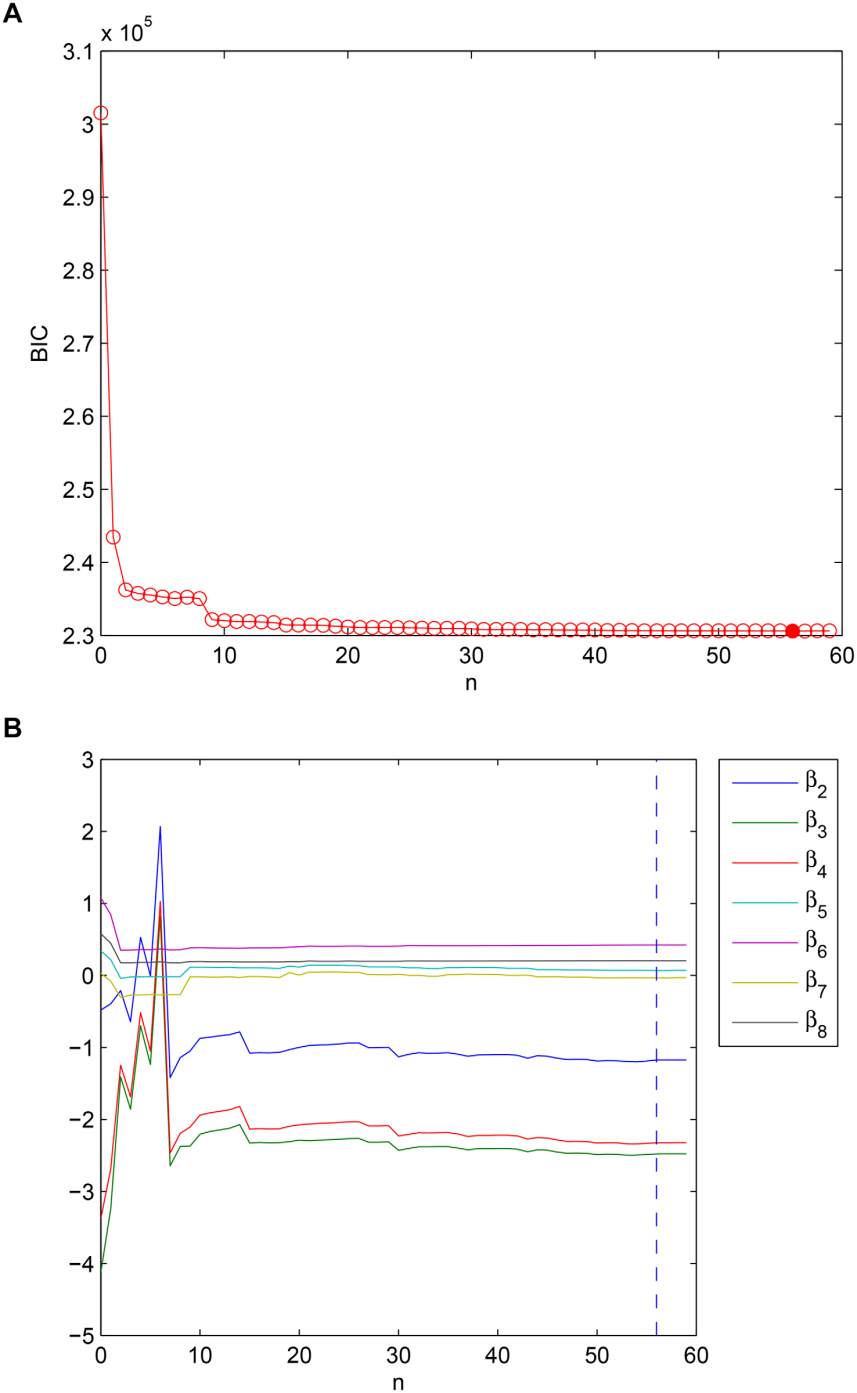
(a) The BICs of models with increasing *n*. (b) The convergence of fix-effect estimates.

**Fig 8:**
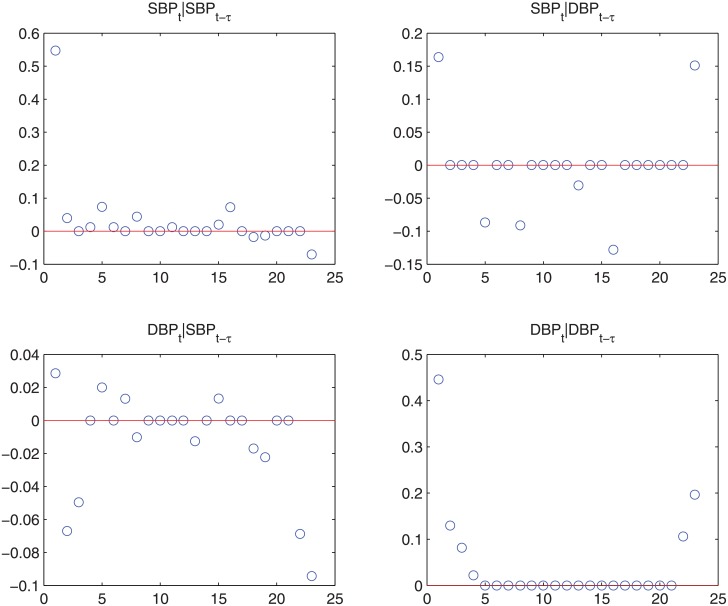
The nonzero entries of *β* showing a circular structure.

**Fig 9:**
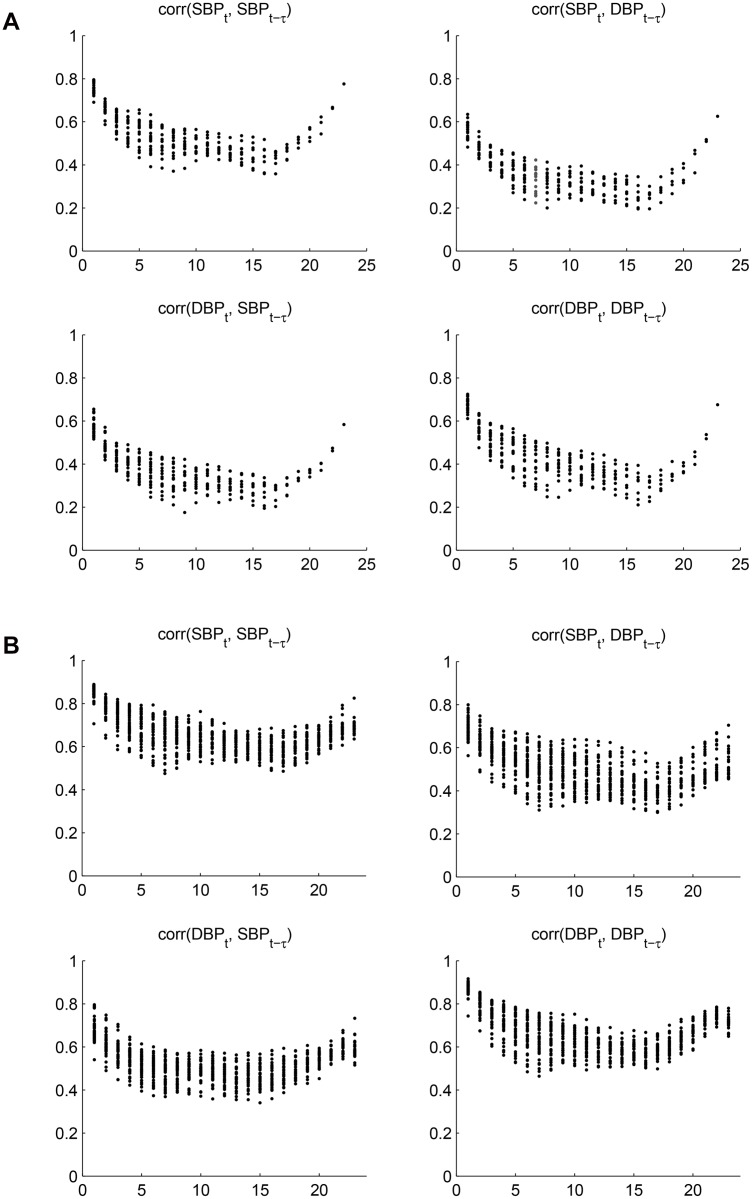
(a) The empirical correlations of SBP and DBP data, where the x-axis is the time lag, *τ*, and each circle represents the correlation between, for example, *SBP*_*t*_ and *DBP*_*t* − *τ*_. Because for a fixed *τ* there can be multiple *t*s depending on the availability of data, we can observe multiple circles at some *τ*.(b) The model-estimated correlations of SBP and DBP data. Because the model estimates are not limited by the availability of data, we have the same number of circles at each *τ*.

From Fig 7(a), we see the minimum BIC appears at *n* = 56 (the actual number of nonzeros in *ρ* is 36), which is far less than the BIC of the LME model; i.e., when *n* = 0. The sharp decline of BIC even at *n* = 1 suggests that adding the AR part to the LME model would be more appropriate. Observing the flattened BIC after *n* = 20, we can choose more parsimonious models. The convergence of fixed-effects estimates is shown in Fig 7(b). Fig 8 shows the estimated *ρ*, and for a better presentation, we split *ρ* into four parts, each at a length of 23. For example, the first subplot shows {*ρ*_11*τ*_∣*τ* = 1,…,23}, which corresponds to the contribution of SBP at time *t* − *τ* to SBP at time *t*. The U-shapes observed in Fig 8 corresponds well to the correlation plots seen in Fig 9(a), which also inspire the circular autoregressive-correlation structure in [14]. We also plot the estimated correlations between predicted BP values in Fig 9(a) with 9(b), and comparing the two figures, we can see that our model can capture more subtle structures such as the W-shapes of the original correlations. The slight elevation of the predicted correlations is due to the removed noise term in the predicted values.

Table 2 presents the estimates, standard errors, and p-values from our SMARLME mode fit, along with those from an LME model fit for comparison with the mean-value model in [13]. The intercept parameter represents the average of SBP and DBP for the non-white, control diet group during the pre-intervention period for the “No AR” model and the residual average after variation removal in the “AR” (SMARLME) model. The “diet group and intervention” parameters indicate the estimated differences in blood pressure from this average for the groups during the intervention period, while the diet parameters indicate these differences during the pre-intervention period. The “White” parameter indicates the estimated difference in blood pressure for White subjects, and the age parameter denotes the estimated change in blood pressure for each yearly increase in age. Clearly, the estimated effects of the DASH diet and the vegetable/fruit diet are both reduced from the mean-value (No AR) model with our (AR) model fit, although they are still significant. Interestingly, even within the control group, there appears to be a significant difference before and after the intervention period with our model fit, as seen in the estimate of the control diet and intervention period, while the mean-value model shows otherwise.

**Table 2:** Estimates of the Mixed-Effects Model.

	No AR			AR		
	Estimate	SE	p-value	Estimate	SE	p-value
Intercept	105.3	1.221	0.000	36.479	0.662	0.000
Control and Intv	-0.481	0.521	0.356	-1.098	0.354	0.002
DASH and Intv	-4.115	0.517	0.000	-2.405	0.356	0.000
Veg/Fruit and Intv	-3.374	0.517	0.000	-2.209	0.357	0.000
DASH	0.34	1.125	0.763	0.103	0.388	0.791
Veg/Fruit	1.076	1.126	0.339	0.411	0.388	0.29
White	0.026	0.907	0.977	0.001	0.315	0.997
Age	0.577	0.212	0.006	0.198	0.073	0.007

## Discussion

The main contribution of this paper is in its (1) explicit modeling of reciprocal features of multiple time series, and (2) offering of a simple and practical solution to the potentially high-dimensional lagged components in the model. There exists a large literature on either components (1) and (2) that could be dated back to early work such as [21]. More recent work in (2) includes the non-reciprocal dynamic-factor model [22], which aims to capture the dynamics of a time series such as a financial indicator with a large number of lagged-predictor variables, such as supply and order variables. Similar to our goal here, different methods such as the principal component and shrinkage method have been proposed to solve the high-dimensional problem [23, 24]. For reciprocal-causal models in (1), earlier work arose both in the psychometric literature, especially in structural equation modeling, and in economics. For example, the so-called cross-lagged models have been developed for reciprocal time series [25], although the method mostly addresses problems of relatively low dimension and short panel of cohort data (in the notation of Eq (1), p = 2 and m = 1). Thus, one can view this paper as a way to extend reciprocal models in time series to high dimensions—both in term of interacting variables and the time variable—and to offer a sparse representation of the model structure. One interesting feature of the proposed method for SMARLME is that it simultaneously addresses the model selection and the estimation problem. Additionally, as we have shown in the circadian oscillation example, nonlinear variations over time can also be modeled using transformed terms in the linear predictive model. Further research is required to evaluate the scope and limit of using linear models for nonlinear feedback systems. Although the current SMARLME modeling setup is such that the sparsity is induced by the cardinality constraint, it is possible that some specific sparse structure could be a priori defined, as pointed out by a reviewer. Such an implementation can indeed limit the FoBa search space and improve computational efficiency.

There are several limitations of the current work. First, we have not addressed stationary conditions of the model. It is possible that the estimated model is non-stationary. However, our focus has been in clinical applications in which the long-term behavior of the model may not be a primary concern. In fact, the FoBa algorithm proposed in this paper does not require that the time series are stationary. A second limitation is that we have not taken into account the impact of model selection on inference [26, 27]. In other words, the selected sparse model structure may not be correct and therefore it is possible that the coefficients and standard errors reported in Table 2 are biased. This is an issue that cannot be adequately covered in this paper. Further research will examine the impact of selecting different sparse model on coverage properties. Finally, a limitation of the current work is that we have restricted the discussion to linear models and avoided nonlinear regression models. The nonlinear circadian rhythm example used for the generative model in our simulation study has been linearized with exponentiated transformed variables. The estimation proceeds using the proposed linear algorithm, which actually brings some simplification to the problem. The simplification could also be useful when interpreting parameters in the fixed and random effect components of SMARLME, which in some cases could be the primary goal of inference, for example in medical applications in which the AR component is treated as a nuisance factor.

## Supporting Information

S1 File S1 File contains an example about the use of matrix formulation for the FoBa estimation of the multivariate autoregressive model. (PDF)
