# Supplementary material for: Analysis of Feedback Mechanisms with Unknown Delay Using Sparse Multivariate Autoregressive Method
Source: PLoS One. 2015 Aug 7;10(8):e0131371. doi: 10.1371/journal.pone.0131371 (PMC4529169; doi:10.1371/journal.pone.0131371)
Supplement: S1 File — (PDF) [file pone.0131371.s001.pdf]

Supplementary Materials

Illustration of matrix representation in estimation method

The following example illustrates the matrix representation of the minimization problem. Here Y_{ijt} is outcome $j, j = 1, \ldots, p$, at time t ,$t = 1, \ldots, T$ for subject $i = 1, \ldots, N$. The general matrix $\boldsymbol{A}$ takes the form:

$$\boldsymbol{A} = \begin{pmatrix} \boldsymbol{I}_p \otimes \boldsymbol{Y}_1 & & \\ \boldsymbol{I}_p \otimes \boldsymbol{Y}_2 & \boldsymbol{I}_p \otimes \boldsymbol{Y}_1 & \\ \boldsymbol{I}_p \otimes \boldsymbol{Y}_3 & \boldsymbol{I}_p \otimes \boldsymbol{Y}_2 & \boldsymbol{I}_p \otimes \boldsymbol{Y}_1 \\ & .... & \\ \boldsymbol{I}_p \otimes \boldsymbol{Y}_{t-1} & ... & \boldsymbol{I}_p \otimes \boldsymbol{Y}_{t-d} \\ & .... & \\ \boldsymbol{I}_p \otimes \boldsymbol{Y}_{T-1} & ... & \boldsymbol{I}_p \otimes \boldsymbol{Y}_{T-d} \end{pmatrix}, \quad (1)$$

where d is the upper limit of the history τ to be included into the model. The $\boldsymbol{\rho}_\tau$ denotes the AR parameter at time lag τ, and $\rho_\tau = (\rho_{kj\tau})$ is the vector of the effect of variable k at time $t - \tau$ on variable j. The vectorization operation of an array by convention uses the rule of first index changes fastest.

This specific example uses $p = 2$ and $N = 3$. For a general time t, we have

$$\boldsymbol{I}_2 \otimes \boldsymbol{Y}_t = \begin{bmatrix} 1 & 0 \\ 0 & 1 \end{bmatrix} \otimes \begin{bmatrix} Y_{11t} & Y_{12t} \\ Y_{21t} & Y_{22t} \\ Y_{31t} & Y_{32t} \end{bmatrix} \quad (2)$$

$$= \begin{bmatrix} Y_{11t} & Y_{12t} & & \\ Y_{21t} & Y_{22t} & & \\ Y_{31t} & Y_{32t} & & \\ & & Y_{11t} & Y_{12t} \\ & & Y_{21t} & Y_{22t} \\ & & Y_{31t} & Y_{32t} \end{bmatrix}. \quad (3)$$

Consider an example of which $T = 3$, $d = 2$, and $q = p = 2$. The residual vector $\boldsymbol{b}$ is given by:

$$\boldsymbol{y} - \boldsymbol{A\rho} = \boldsymbol{y} - \begin{pmatrix} \boldsymbol{I}_2 \otimes \boldsymbol{Y}_1 & \\ \boldsymbol{I}_2 \otimes \boldsymbol{Y}_2 & \boldsymbol{I}_2 \otimes \boldsymbol{Y}_1 \end{pmatrix} \begin{pmatrix} \boldsymbol{\rho}_1 \\ \boldsymbol{\rho}_2 \end{pmatrix} \quad (4)$$

$$= \begin{bmatrix} Y_{112} \\ Y_{212} \\ Y_{312} \\ Y_{122} \\ Y_{222} \\ Y_{322} \\ Y_{113} \\ Y_{213} \\ Y_{313} \\ Y_{123} \\ Y_{223} \\ Y_{323} \end{bmatrix} - \begin{bmatrix} Y_{111} & Y_{121} & & & & & & \\ Y_{211} & Y_{221} & & & & & & \\ Y_{311} & Y_{321} & & & & & & \\ & & Y_{111} & Y_{121} & & & & \\ & & Y_{211} & Y_{221} & & & & \\ & & Y_{311} & Y_{321} & & & & \\ Y_{112} & Y_{122} & & & Y_{111} & Y_{121} & & \\ Y_{212} & Y_{222} & & & Y_{211} & Y_{221} & & \\ Y_{312} & Y_{322} & & & Y_{311} & Y_{321} & & \\ & & Y_{112} & Y_{122} & & & Y_{111} & Y_{121} \\ & & Y_{212} & Y_{222} & & & Y_{211} & Y_{221} \\ & & Y_{312} & Y_{322} & & & Y_{311} & Y_{321} \end{bmatrix} \begin{bmatrix} \rho_{111} \\ \rho_{211} \\ \rho_{121} \\ \rho_{221} \\ \rho_{112} \\ \rho_{212} \\ \rho_{122} \\ \rho_{222} \end{bmatrix}.$$

As an example, the residual corresponds to outcome 2 of subject 1 at time 3, Y_{123}, is predicted by the following linear function:

$$\rho_{121}Y_{112} + \rho_{221}Y_{122} + \rho_{122}Y_{111} + \rho_{222}Y_{121}, \quad (5)$$

of which the components correspond to contributions from the historical effects from both the first and second outcomes of the same subject 1. For example, $\rho_{121}Y_{112}$ is the contribution from outcome variable 1 of lag 1 on outcome variable 2 at time 3, or Y_{123} in this case. Note that in the circadian example, both $\exp(Y_{ik(t-\tau)})$ and $Y_{ik(t-\tau)}$ were used as predictors. In our actual estimation, we used a slight modification of the matrix $\boldsymbol{A}$ by eliminating the rows that contained zeros on the right upper corner of $\boldsymbol{A}$ in equation (1). When the sample size is large and the number of time points T is large relative to d, the modified matrix A generally leads to reduced bias in the estimate of $\boldsymbol{\rho}$.
